# QTL mapping and genomic prediction of resistance to apple blotch (*Diplocarpon coronariae*)

**DOI:** 10.1007/s11032-026-01666-5

**Published:** 2026-05-02

**Authors:** Michaela Jung, Bettina Hänni, Hélène Muranty, Andrea Patocchi

**Affiliations:** 1 Agroscope, Mueller-Thurgau-Strasse 29, Waedenswil, 8820 Switzerland; 2 Fructus, Mueller-Thurgau-Strasse 29, Waedenswil, 8820 Switzerland; 3 Univ Angers, Institut Agro, INRAE, IRHS, SFR QuaSaV, Angers, F-49000 France

**Keywords:** Marssonina leaf blotch, *Diplocarpon coronariae*, Disease resistance, Breeding

## Abstract

Apple blotch (AB) is an emerging disease in apple production, primarily causing premature defoliation. The rise of orchard management practices reducing fungicide use has coincided with an increase in AB incidence in Europe. Breeding for resistance to AB became important for long-term disease management. Depending on the genetic complexity of the trait, breeding can be supported using either marker-assisted or genomic selection. However, no such tools are currently available for AB. To address this issue, we established two biparental mapping populations, ‘Topaz’ × ‘Granny Smith’ and ‘Topaz’ × ‘Parkapfel’, and a diversity panel of 122 genotypes, all evaluated for their resistance to AB under natural field conditions across three growing seasons. Quantitative trait locus (QTL) mapping identified five associated loci on chromosomes 3, 5, 6, 10, and 11, each explaining a maximum of 12% of phenotypic variance, indicating a polygenic architecture of AB resistance. Strong seasonal effects were observed in disease development, and they were either removed (G model) or explicitly modeled (G + G×E model) using genomic prediction approaches. The average predictive ability reached 0.47 for the G model and 0.38 for the G + G×E model. While the detected QTLs have small individual effects that constrain their use in marker-assisted selection, the moderate predictive ability of genomic prediction models demonstrates the potential of genomic selection for accelerating resistance breeding. These findings provide the first genomic tools to support breeding for resistance to AB and illustrate the utility of genome-wide approaches in managing complex traits.

**Supplementary Information:**

The online version contains supplementary material available at 10.1007/s11032-026-01666-5.

## Introduction

Apple blotch (AB), also known as apple Marssonina blotch or Marssonina leaf blotch, is a disease caused by the fungus *Diplocarpon coronariae* (Ellis & Davis) Wöhner & Rossman, formerly known as *Marssonina coronaria* (Ellis & Davis) Davis. Typical early symptoms of AB include small, circular brown to black spots with acervuli on the upper surface of mature leaves. As the infection progresses, star-shaped necrotic lesions also containing acervuli develop, often leading to leaf yellowing and premature leaf drop. In rare cases, black spots may also appear on the fruit (Wöhner and Emeriewen 2019).

Distributed mainly in Asia, the disease has been spreading in Europe since the early 2000s, primarily affecting orchards that use biological or no plant protection (Tamietti and Matta 2003; Persen et al. 2012; Hinrichs-Berger and Müller 2013). In addition to the reduction of fungicide use, the infection is promoted by warm and humid conditions (Lian et al. 2021), potentially accelerating the future spread of AB under changing climatic conditions. For long-term disease management, breeding for disease resistance represents a viable strategy. However, most apple cultivars, including ‘Topaz’, one of the most widely adopted apple scab-resistant cultivars in Europe, have been described as susceptible to AB (Wöhner and Emeriewen 2019; Hinrichs-Berger and Müller 2013). Cultivating apple scab-resistant cultivars can allow reducing fungicide use, provided they are also resistant to AB. The limited availability of cultivars resistant to AB that can serve as effective resistance donors in breeding programs represents a substantial constraint. Nevertheless, several genotypes exhibiting partial resistance or reduced symptom expression have been identified (Richter et al. 2024; Sharma et al. 2011; Noh et al. 2020). Among the tested germplasm, the cultivar ‘Granny Smith’ has exhibited reduced symptoms of AB under both artificial inoculation and natural field conditions (Sharma et al. 2011; Rather et al. 2017; Noh et al. 2020). These descriptions of both susceptible and partially resistant cultivars can inform genetic studies and help identify resistance-associated loci.

Genome-wide association studies (GWAS) have already identified multiple loci associated with AB disease resistance and symptom development, indicating a polygenic architecture and several candidate genes involved in resistance (Richter et al. 2025; Noh et al. 2020). While Richter et al. (2025) performed repeated laboratory inoculation experiments, Noh et al. (2020) studied the severity of AB in an open orchard during one season. These studies benefited from existing germplasm collections, thereby circumventing the time and resource investment required to develop dedicated mapping populations. However, populations derived from crosses between susceptible and (partially) resistant cultivars could be used for quantitative trait locus (QTL) mapping, offering complementary insights to GWAS and ultimately enabling the identification of genetic markers for application in marker-assisted selection. If multiple associated markers were identified across analyses, indicating genetic complexity of a trait, a genome-wide approach of genomic selection (Meuwissen et al. 2001) may represent a more effective alternative to marker-assisted selection. Genomic selection relies on training populations that are both phenotyped and genotyped to develop genomic prediction models, which can then be applied to individuals requiring only genotypic data, thereby reducing the need for extensive phenotyping and increasing breeding efficiency.

This study aimed to evaluate AB resistance and conduct QTL mapping in the progeny from two crosses involving the susceptible cultivar ‘Topaz’ and the partially resistant parents ‘Granny Smith’ and ‘Parkapfel’. The latter is a genetic resource chosen as the parent due to its reduced level of infection with AB, demonstrated under field conditions in Switzerland by the absence of symptoms in four trees grown at two sites over two consecutive years (FRUCTUS, unpublished). To validate and complement the QTL mapping outcomes, an additional AB resistance evaluation was performed on a diversity panel. For the first time, the AB disease and defoliation traits were repeatedly evaluated under field conditions over the course of three seasons, with the aim of achieving robust phenotypic assessment that accounted for environmental effects. The ultimate goal was twofold: (i) to identify genetic markers associated with AB resistance for potential use in marker-assisted selection, and (ii) to evaluate the suitability of genomic selection as an alternative strategy for improving AB disease resistance.

## Methods

### Plant material

Progenies from two crosses were grown in Waedenswil, Switzerland, since March 2021. The full-sib progeny from the ‘Topaz’ × ‘Granny Smith’ cross comprised 163 genotypes while the full-sib progeny from the ‘Topaz’ × ‘Parkapfel’ cross consisted of 168 genotypes. The genotypes were not clonally replicated but grafted onto the ‘M9’ rootstock. The MUNQ (Malus UNiQue genotype code), as described by Muranty et al. (2020) and Durel et al. (2023), was 1213 for ‘Topaz’, 548 for ‘Granny Smith’, and 3592 for ‘Parkapfel’.

A diversity panel was cultivated in Waedenswil, Switzerland, since 2016. The 122 genotypes included in this study were chosen from a larger population of Swiss genetic resources (cultivated apple accessions only, i.e., no wild apple species included), with their number limited by the availability of genomic data. A list of these genotypes, including their MUNQ and the number of replicates phenotyped per genotype each year (ranging from one to eight), can be found in Online Resource 1. The majority of genotypes (91) were replicated twice, with each replicate assigned to one of two different blocks. The genotypes were grafted onto the ‘M9’ rootstock using the interstem ‘Golden Delicious’ or ‘Schneiderapfel’. The susceptible genotype ‘Golden Delicious’ served as a control and was replicated 58 times throughout the orchard. All genotypes studied were cultivated without the application of fungicides during the assessment period.

## Phenotyping

The assessment scale for the severity of AB disease (hereafter referred to as “disease”) and the defoliation due to AB (hereafter referred to as “defoliation”) was adapted from Lateur and Populer (1994) as shown in Table 1. Disease assessments for both the ‘Topaz’ × ‘Granny Smith’ and ‘Topaz’ × ‘Parkapfel’ crosses were conducted on up to three timepoints (T1–T3) in 2022, 2023, and 2024 (Table 2). Defoliation was estimated for both crosses on up to three timepoints (T1–T3) in 2023 and 2024 (Table 2).

The diversity panel was inoculated with AB by distributing 800 bags containing infected leaf material throughout the orchard in October 2020. Disease assessments were conducted once per year as shown in Table 2. Trees with substantial leaf fall but only limited AB symptoms were assigned missing values.

**Table 1:** Assessment scale for disease and defoliation due to AB. Intermediate grades are provided to support situations where it is difficult to choose between two grades

Grade	Symptoms	Leaves affected	
		Disease	Defoliation
1	Not visible	0%	0%
2	A few lesions or spots detectable on close inspection, absence of yellow leaves		
3	A few infected leaves immediately apparent, first yellow leaves may appear	1–5% (attached leaves)	1–5% (fallen leaves)
4	Intermediate		
5	Widespread infection, first fallen leaves may appear	~ 25% (attached or fallen leaves)	~ 25% (fallen leaves)
6	Intermediate		
7	Severe infection, about half of the leaves show symptoms or have fallen	~ 50% (attached or fallen leaves)	~ 50% (fallen leaves)
8	Intermediate	~ 75% (attached or fallen leaves)	~ 75% (fallen leaves)
9	Nearly all leaves show symptoms or have fallen	> 90% (attached or fallen leaves)	> 90% (fallen leaves)

**Table 2:** Timepoints (T1–T3) of disease assessment defined as day/month for the crosses ‘Topaz’ × ‘Granny Smith’ (C1), ‘Topaz’ × ‘Parkapfel’ (C2), and the diversity panel (DP). The timepoints at which defoliation was assessed are marked with an asterisk (*)

Year	T1		T2		T3		
	C1	C2	C1	C2	C1	C2	DP
2022	-	26/08	12/09	12/09	04/10	04/10	29/09
2023	01/09	01/09	25/09*	25/09*	06/10*	06/10*	25–26/09
2024	30/08*	30/08*	18/09*	18/09*	07/10*	07/10*	2–3/10

## Genotyping

SNP genotyping and filtering was performed for the progenies and their parents, ‘Topaz’, ‘Granny Smith’ and ‘Parkapfel’, as described by Di Pierro et al. (2016) at the Fondanzione Edmund Mach, Italy. Briefly, the SNPs were obtained using the Illumina Infinium^®^ 20 K SNP genotyping array (Bianco et al. 2014) and filtered with ASSIsT (Di Guardo et al. 2015). SNPs that were identified as robust by ASSIsT were retained. Genotypes identified by ASSIsT as outcrossings were excluded (three genotypes from the ‘Topaz’ × ‘Granny Smith’ cross and twelve genotypes from the ‘Topaz’ × ‘Parkapfel’ cross). ASSIsT did not identify any further genotypes for removal due to different ploidy levels or DNA admixture. In addition to filtering by ASSIsT, any SNPs that were heterozygous in both parents of each progeny were removed. After filtering, the missing data accounted for 0.06 and 0.04% in the ‘Topaz’ × ‘Granny Smith’ and ‘Topaz’ × ‘Parkapfel’ crosses, respectively. These missing values for each cross were replaced with the mean allele dosage of the respective SNPs, rounded to the nearest integer. Finally, a set of 4,193 SNPs was obtained for 160 genotypes of the ‘Topaz’ × ‘Granny Smith’ cross, and 3,883 SNPs were acquired for 156 genotypes of the ‘Topaz’ × ‘Parkapfel’ cross. The physical positions of the SNPs were based on the doubled haploid GDDH13 (v1.1) reference genome (Daccord et al. 2017) following the updated iGL map (Howard et al. 2021b). The maximum physical distances between adjacent SNPs on each chromosome, including the terminal regions, were determined. Chromosome ends were defined by the SNPs with the maximum genomic position in the 303 K SNP dataset reported by Jung et al. (2020). To enable a comparison of allelic effects between the crosses and the diversity panel, the strand orientation was corrected using the plugin “fixref” of the BCFtools (v1.21) (Danecek et al. 2021).

For the diversity panel, the genomic data of 81 genotypes at the resolution of the Axiom^®^ Apple 480 K array (Bianco et al. 2016) were retrieved from previous studies (Urrestarazu et al. 2017; Muranty et al. 2020). The remaining 41 individuals genotyped by the Illumina Infinium^®^ 20 K SNP genotyping array (Bianco et al. 2014) were obtained from earlier studies (Howard et al. 2021a, b, 2023; Larsen et al. 2024, 2025; Kumar et al. 2021). The physical positions of all SNPs were based on the doubled haploid GDDH13 (v1.1) reference genome (Daccord et al. 2017). Genomic data from the 480 K SNP array were available for 303,237 SNPs, and the 20 K SNP array dataset contained 7,063 SNPs that shared physical positions with the 480 K SNP array dataset. The missing SNP values in the 20 K SNP array dataset were imputed using a set of accessions and pedigrees from previous studies (Urrestarazu et al. 2017; Howard et al. 2018; Muranty et al. 2020) as described by Jung et al. (2020). Prior to the imputation, all genomic data used were subjected to the correction of strand orientation applying the plugin “fixref” of the BCFtools (v1.21) (Danecek et al. 2021). The final genomic dataset for the diversity panel contained 303,237 SNPs for 122 individuals.

## Phenotypic data analysis

For the progenies from the two crosses, disease and defoliation traits were used to calculate the maximum score (MAX) for each year, based on the scores at each timepoint within that year. The area under the disease progress curve (AUDPC) was also calculated annually across timepoints using the agricolae (v1.3-7) R package (de Mendiburu and Yaseen 2020). For each of these summary traits, disease MAX, disease AUDPC, defoliation MAX, and defoliation AUDPC, a mixed-effects model was fitted using the lme4 (v1.1-37) R package (Bates et al. 2015) as:

1 $$\boldsymbol{y}=\mathbf{X}\boldsymbol{\beta\:}+\:\mathbf{Z}\boldsymbol{u}+\boldsymbol{\epsilon}$$

where $\:\boldsymbol{y}$ is the vector of the phenotypic values, $\:\:\mathbf{X}$ the fixed-effects design matrix, $\:\boldsymbol{\beta\:}$ the vector of fixed effects, $\:\mathbf{Z}$ the random-effects design matrix, $\:\boldsymbol{u}$ the vector of random effects and $\:\boldsymbol{\epsilon\:}$ the vector of random errors assuming normal distribution with zero mean and variance $\:{\sigma\:}_{\epsilon\:}^{2}$. The mixed-effects model described in Eq. 1 included genotype as a fixed effect and year as a random effect. This model was used to obtain fixed-effect estimates, also known as best linear unbiased estimates (BLUEs). The summary traits were used to fit an additional mixed-effects model according to Eq. 1 (later referred to as the genetic model), with genotype and year as independent random effects, to estimate the variance components for these effects. The disease and defoliation traits from all timepoints were scaled and centered to perform a principal components analysis and visualize the output in a biplot.

For the disease trait of the diversity panel, a mixed-effects model following Eq. 1 was fitted with genotype as a fixed effect and year, genotype × year interaction, genotype replicate (i.e., tree), and block as random effects. The BLUEs for the fixed effect of genotype were extracted from the model fit. Year-specific disease BLUEs were estimated using the mixed-effects model in Eq. 1, with genotype fitted as a fixed effect and block as a random effect, modeled separately for each year. To estimate the variance components for all model terms, an additional mixed-effects model according to Eq. 1, i.e., the genetic model, was fitted with the random effects of genotype, year, genotype × year interaction, genotype replicate, and block.

## QTL mapping

For each combination of the two crosses, four summary traits, and the available SNPs, a Kruskal–Wallis rank sum test was conducted using the BLUEs as the response variable and SNP allele dosage as the grouping variable. The Bonferroni-corrected significance threshold was calculated as $\:{\alpha\:}^{*}=\alpha\:/m$, where $\:{\upalpha\:}\:=\:0.05$ and $\:m$ is the total number of SNPs. Multiple-testing correction was additionally performed using a permutation-based approach: the BLUEs were randomly shuffled (i.e., permuted), and the Kruskal–Wallis test was repeated for each of 10,000 permutation replicates. For each permutation replicate, the genome-wide minimum *p*-value was recorded. The significance threshold was set as the 5th percentile of these minimum *p*-values. SNP markers positioned at the top of prominent peaks in the Manhattan plot, either significantly associated based on the permutation-based approach or nearing the permutation-based threshold, were hereafter referred to as QTLs.

The proportion of phenotypic variance explained by the individual QTLs was estimated for each summary trait using a mixed-effects model following Eq. 1 that included random effects for genotype, year and, where applicable, each QTL (later referred to as the QTL model).

Multi-locus allelic combinations were defined by concatenating the allelic combinations of the individual QTLs: (i) in the summary trait where more than one QTL was detected in a cross, and (ii) in the summary trait where more than one QTL was detected between crosses and the associated SNPs identified using QTL mapping were present in the genomic dataset for the diversity panel. For (i), trait and SNP information of the cross was used, while in case of (ii), the disease trait and the SNPs found in the diversity panel were used. Separately for (i) and (ii), a likelihood ratio test was then performed for the mixed-effects model following Eq. 1, which included a fixed effect of the multi-locus allelic combination × year interaction and a random effect of genotype. If the interaction was not significant, the effects of multi-locus allelic combination and year were tested separately.

### Haplotype blocks

Haplotype blocks were estimated for the diversity panel following Gabriel et al. (2002) as implemented in PLINK (v.1.9-beta6.27) (Chang et al. 2015; Purcell & Chang n.d.). Pairs of SNPs within 10 Mb from each other were considered. SNPs with a minor allele frequency of less than 0.05 were excluded. When the associated SNPs identified using QTL mapping were not present in the genomic dataset for the diversity panel, haplotype blocks were assigned to QTLs based on the physical positions of the corresponding SNPs within those blocks. If no haplotype block could directly be assigned to a QTL, the SNP with the next lowest *p*-value that could be assigned to a haplotype block on the same chromosome was used instead. All SNPs within the assigned haplotype blocks were collapsed into multi-SNP combinations for each genotype and QTL. For the disease trait of the diversity panel, a mixed-effects model according to Eq. 1 (i.e., the QTL model) was fitted to estimate the proportion of phenotypic variance explained by the QTLs represented by SNPs when available, or otherwise by haplotype blocks. This model included random effects for genotype, year, genotype × year interaction, genotype replicate, block, and each QTL.

## Genomic prediction and genomic heritability

Genomic predictions for the diversity panel were performed using the Bayesian ridge regression model (later termed the G model) defined as:

2 $$\boldsymbol{y}=\mu\:+\mathbf{M}\boldsymbol{u}+\boldsymbol{\epsilon}$$

where $\:\boldsymbol{y}$ is the response vector of disease BLUEs, $\:\mu\:$ is the intercept, $\:\mathbf{M}$ is the design matrix of centered and standardized SNP markers, $\:\boldsymbol{u}$ is the vector of random genomic effects, and $\:\boldsymbol{\epsilon\:}$ is the vector of random errors assuming normal distribution with zero mean and residual variance $\:{\sigma\:}_{\epsilon\:}^{2}$.

The multi-environment genomic prediction model (referred to as the G + G×E model) following Lopez-Cruz et al. (2015) that applied the reproducing kernel Hilbert spaces regression was defined as:

3 $$y=\boldsymbol{\mu\:}+\mathbf{Z}\boldsymbol{u}+\boldsymbol{\epsilon}$$

where $\:\boldsymbol{y}$ is the response vector of year-specific disease BLUEs, $\:\boldsymbol{\mu\:}$ is the vector with an intercept for each year, $\:\mathbf{Z}$ is the design matrix for random effects, $\:\boldsymbol{u}$ is the vector of random effects, and $\:\boldsymbol{\epsilon\:}$ is the vector of random errors assuming $\boldsymbol{\epsilon}\sim N(0,{\mathbf I\sigma_\epsilon^2})$, where $\:\mathbf{I}$ is the identity matrix. The vector $\:\boldsymbol{u}={\boldsymbol{u}}_{0}+{\boldsymbol{u}}_{1}$, where $\:{\boldsymbol{u}}_{0}$ is the vector of random main genomic effects (common to all years) and $\:{\boldsymbol{u}}_{1}$ is the vector of genomic × year interaction effects (random deviations of the genomic effects for specific years). The vector $\;{\boldsymbol {u}_0}\sim N(0,\;{{\mathbf G}_0\sigma}_{\boldsymbol{u}\mathbf{0}}^2)$, where $\:{\mathbf{G}}_{0}=\mathbf{J}\otimes\:\mathbf{G}$, with $\:\otimes\:$ denoting the Kronecker product. Here, $\:\mathbf{J}$ is a matrix of ones of dimension $t\times\:t$, with $\:t$ being the number of years. The matrix $\mathbf{G}=\mathbf{M}{\mathbf{M}}^{\mathbf{{\prime}}}/p$, with $\:p$ being the number of SNP markers. The vector ${\boldsymbol {u}_1}\boldsymbol\sim {N}(0,\;{\mathbf G}_1)$ with

$$\:{\mathbf{G}}_{1}=\left[\begin{array}{ccc}{\mathbf{G}\sigma\:}_{u1}^{2}&\:0&\:0\\\:0&\:{\mathbf{G}\sigma\:}_{u2}^{2}&\:0\\\:0&\:0&\:{\mathbf{G}\sigma\:}_{u3}^{2}\end{array}\right]$$

        

where $\:\mathbf{G}$ is as described previously. The G and G + G×E models were applied with 12,000 iterations of the Gibbs sampler, a thinning of five, and a burn-in of 2,000 discarded samples using the BGLR (v1.1.4) R package (Pérez and de los Campos 2014). The models were fitted via 10-fold cross-validation, with the process repeated ten times using different seeds. Each fold was based on subsampling 90% of the genotypes without replacement. The predictive ability for the G model was estimated as the Pearson correlation coefficient between disease BLUEs and predicted values across the validation sets for each cross-validation repetition, resulting in ten values of predictive ability. For the G + G×E model, predictive ability was estimated separately for each year as the Pearson correlation between the observed year-specific disease BLUEs and the corresponding predicted values across the validation sets for each cross-validation repetition, resulting in 30 values of predictive ability.

To estimate the proportion of phenotypic variance explained by genomic effects for the G model, and the proportion of phenotypic variance explained by genomic and genomic × year interaction effects for the G + G×E model, these models were applied to the full set of genotypes without cross-validation. Using the model G, genomic heritability was calculated as $\:{h}^{2}={V}_{g}/\left({V}_{g}+{V}_{e}\right)$, where $\:{V}_{g}$ is the genomic variance (i.e., phenotypic variance explained by the genomic effects) and $\:{V}_{e}$ is the residual variance (i.e., phenotypic variance unexplained by the genomic effects). The genomic variance was estimated following the M2 method described by Lehermeier et al. (2017).

## Results

### Phenotypic data characterization

**Fig. 1:**
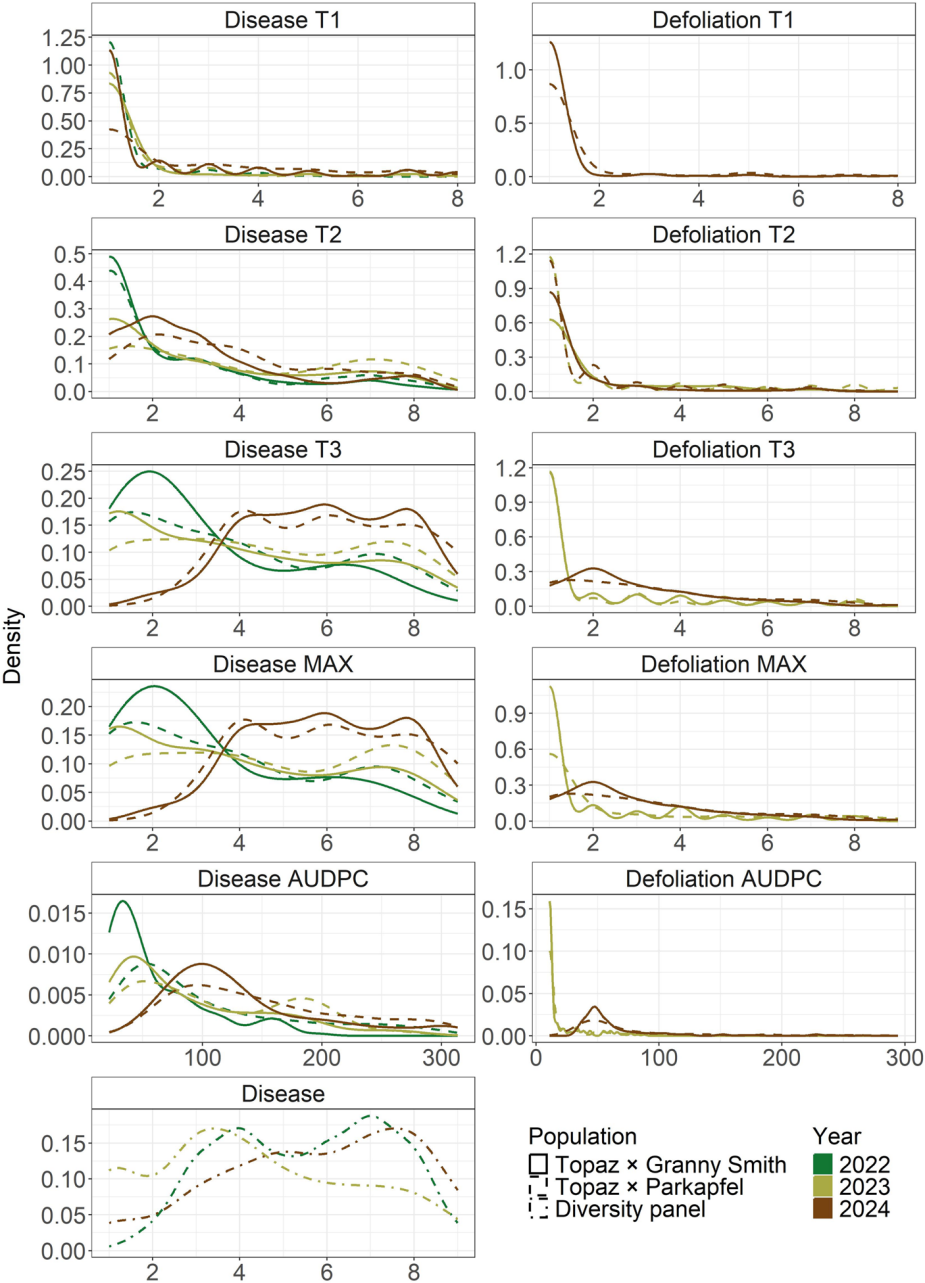
Distributions of phenotypic traits across years and populations studied. The traits were assessed at up to three timepoints (T1–T3) each year. From these data, the maximum score (MAX) and the area under the disease progress curve (AUDPC) were calculated

Various distribution patterns were observed for the studied traits (Fig. 1). For disease-related traits, the distribution shifted from left-skewed at the timepoint T1 to multimodal at T3, reflecting the seasonal progression of disease symptom development. This progression was ultimately reflected in the distributions for the disease AUDPC trait. Disease scores at T3 and the maximum disease scores (disease MAX) in the crosses, as well as the disease trait in the diversity panel, were recorded at the same or similar time points and resulted in comparable distribution shapes. Over the years, both crosses exhibited a gradual shift in phenotypic values toward higher severity classes. In 2022, the lowest severity classes (1 and 2) were more prevalent in the ‘Topaz’ × ‘Granny Smith’ cross compared to the ‘Topaz’ × ‘Parkapfel’ cross. However, in subsequent years, the distributions for both crosses became more similar in shape. For defoliation traits, the absence of highly defoliated genotypes resulted in consistently left-skewed distributions across years and crosses.

**Fig. 2:**
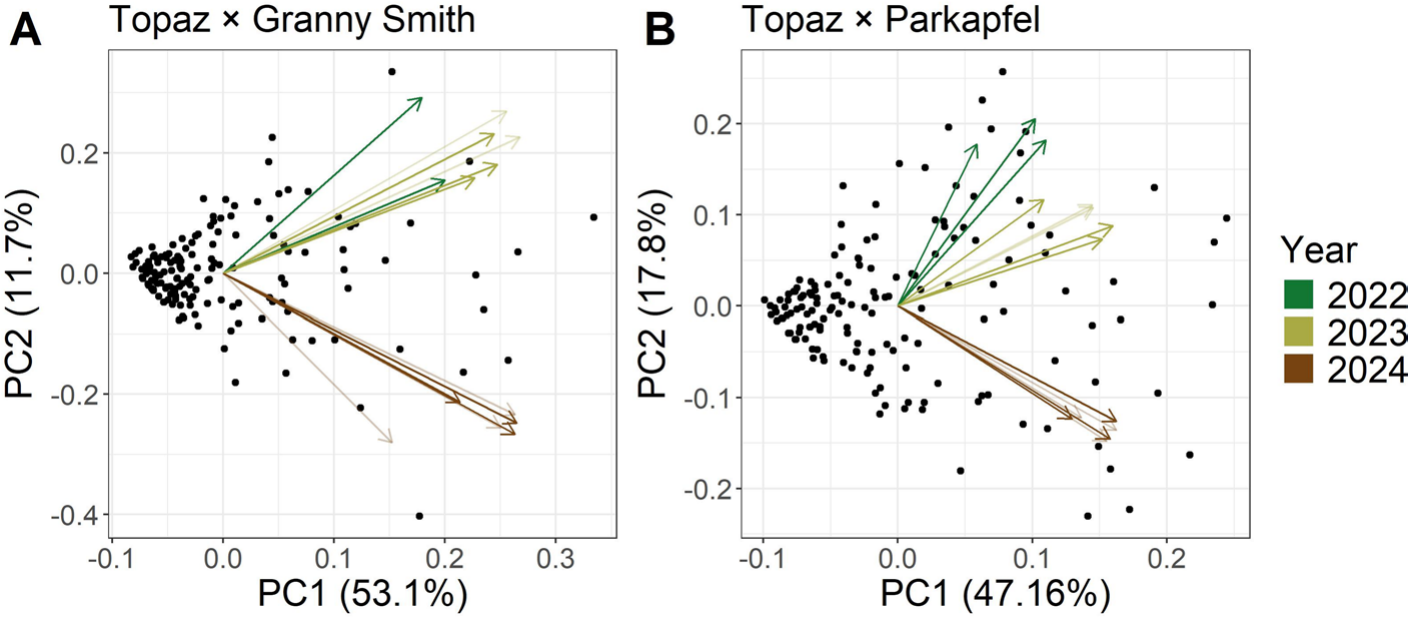
Principal component (PC) analysis biplots based on disease and defoliation traits assessed at up to three timepoints each year, showing trait loadings as arrows colored by the year of measurement. Darker and lighter color shades represent disease and defoliation traits, respectively. Panels A and B display the results for the crosses ‘Topaz’ × ‘Granny Smith’ and ‘Topaz’ × ‘Parkapfel’, respectively

PCA of disease and defoliation traits assessed at up to three timepoints per year (disease T1–T3, defoliation T1–T3) revealed that the first two principal components accounted for a similar proportion of the total variance of 64.80% and 64.96% in the ‘Topaz’ × ‘Granny Smith’ and ‘Topaz’ × ‘Parkapfel’ crosses, respectively (Fig. 2). In both crosses, the angles between trait loadings indicated a stronger positive correlation between disease and defoliation traits in 2022 and 2023 compared to 2024, reflecting a difference in phenotypic traits between 2024 and the earlier years.

### Identified quantitative trait loci

**Fig. 3:**
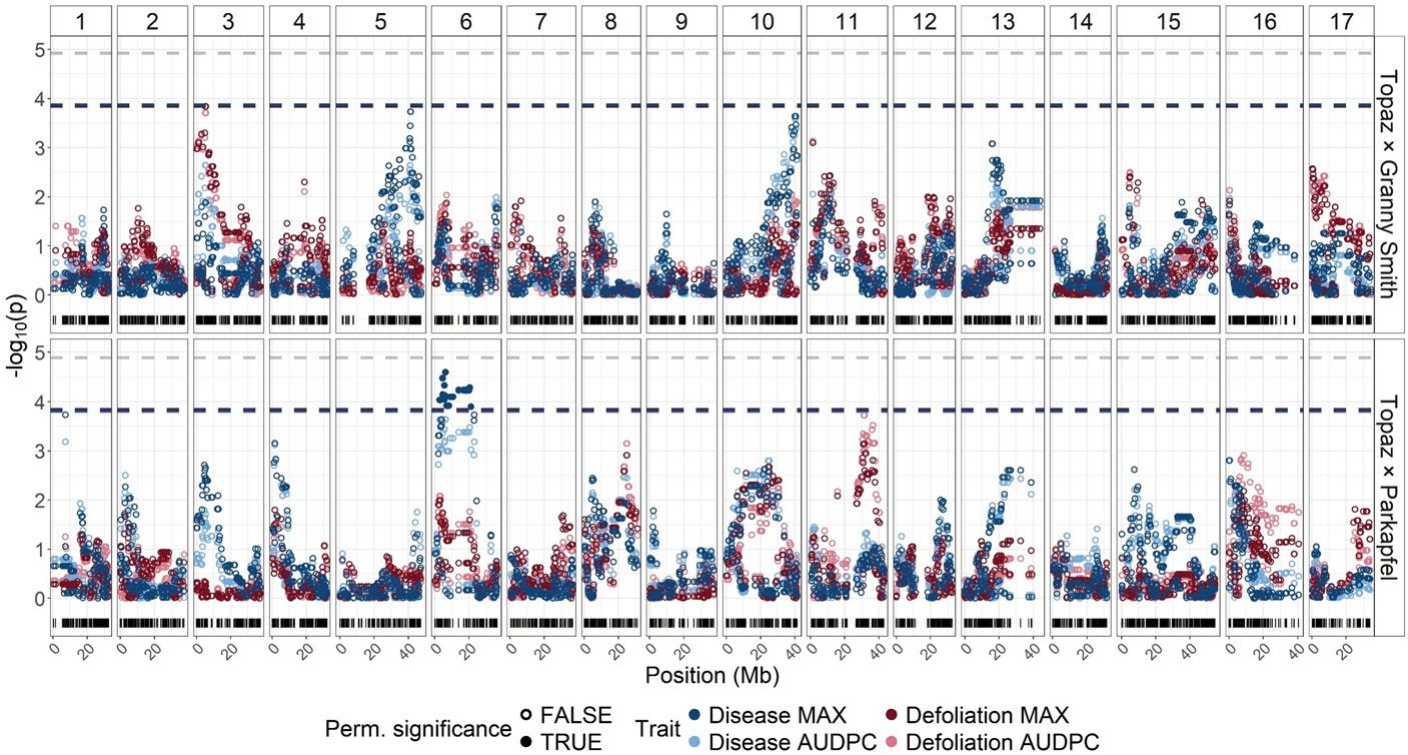
Results of QTL mapping for the two crosses ‘Topaz’ × ‘Granny Smith’ (top panel) and ‘Topaz’ × ‘Parkapfel’ (bottom panel) as well as four summary traits (indicated by color). Trait-specific significance thresholds, determined by permutation testing, are shown as overlapping dashed lines in the corresponding colors. The grey dashed line represents the Bonferroni-corrected significance threshold. The distribution of SNP physical positions along the chromosomes is shown using black vertical bars along the x-axes

Among the 4,193 SNPs retained for the ‘Topaz’ × ‘Granny Smith’ cross, the largest physical interval lacking SNP coverage was 9.17 Mb on chromosome 5 (Online Resource 2). Within the 3,883 SNPs of the ‘Topaz’ × ‘Parkapfel’ cross, the largest SNP-free physical interval was 7.11 Mb on chromosome 12. QTL mapping for both crosses and four summary traits identified five distinct QTLs (Fig. 3, Online Resource 3). The first three QTLs were found in the ‘Topaz’ × ‘Granny Smith’ cross, with ‘Granny Smith’ being the heterozygous parent. QTL1 was associated with the traits defoliation MAX and defoliation AUDPC and was represented by a SNP on chromosome 3 at 5.18 Mb (SNP_FB_0513959). QTL2, associated with disease MAX, was located on chromosome 5 at 41.35 Mb (RosBREEDSNP_SNP_GA_8684631_Lg5_01942_MAF10_327965_exon12). QTL3 was associated with disease MAX and disease AUDPC, and it was represented by a SNP located on chromosome 10 at 40.09 Mb (SNP_FB_0050205). The remaining two QTLs were identified in the ‘Topaz’ × ‘Parkapfel’ cross, with ‘Topaz’ representing the heterozygous parent. QTL4 was associated with disease MAX and disease AUDPC, and it mapped to chromosome 6 at 6.19 Mb (SNP_FB_0655287). QTL5 was associated with defoliation AUDPC and located on chromosome 11 at 31.28 Mb (SNP_FB_0086989).

Of the five QTLs, the QTL4 showed maximum $\:-{log}_{10}\left(p\right)$ of 4.59, which exceeded the significance threshold determined by permutation testing. Depending on the specific cross and trait, the significance thresholds ranged from 0.00013 to 0.00016 (corresponding to $\:-{log}_{10}\left(p\right)$ values between 3.87 and 3.81). The remaining four QTLs did not surpass these significance thresholds but showed maximum $\:-{log}_{10}\left(p\right)$ of 3.84 for QTL1, 3.73 for QTL2, 3.64 for QTL3, and 3.72 for QTL5. None of the identified QTLs reached the Bonferroni-corrected significance threshold.

**Fig. 4:**
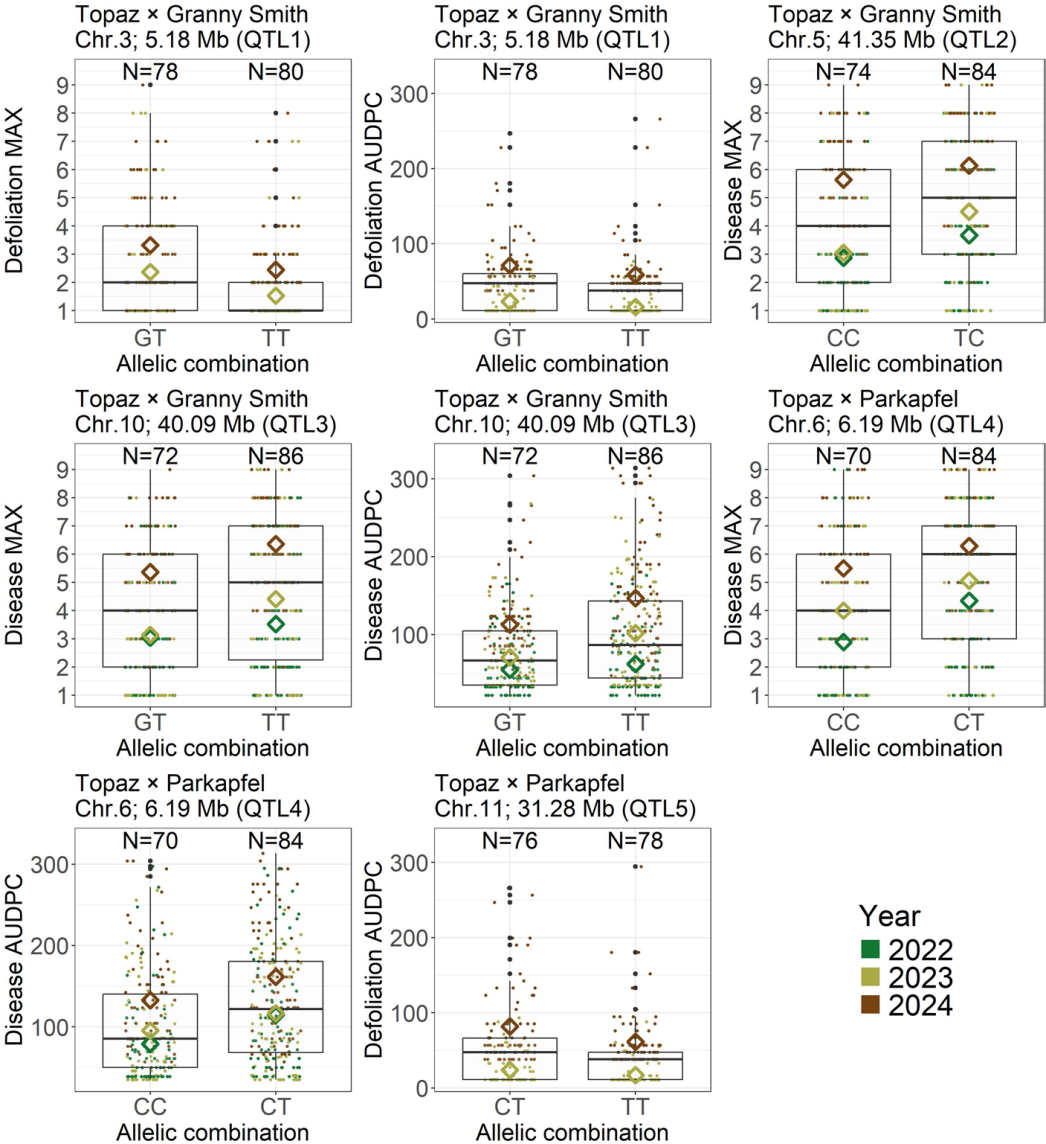
Boxplots showing the distribution of summary traits across years and allelic combinations for the associated QTLs. Boxes represent the interquartile range, with the median indicated by a horizontal line. Whiskers extend to 1.5 times the interquartile range, and black points outside this range represent outliers. The jittered points show the phenotypic values for each genotype, colored according to the year of measurement. Diamonds represent the mean value for each year across genotypes. The number of genotypes for each allelic combination is shown above the corresponding box

Per QTL and allelic combination, an increasing trend in AB symptom incidence was evident in the year-specific mean phenotypic values, with symptom severity rising over successive years (Fig. 4). Across years, the difference in mean phenotypic value between allelic combinations was 0.86 for QTL1 and defoliation MAX, 9.84 for QTL1 and defoliation AUDPC, 0.92 for QTL2 and disease MAX, 0.92 for QTL3 and disease MAX, 24.28 for QTL3 and disease AUDPC, 1.12 for QTL4 and disease MAX, 28.84 for QTL4 and disease AUDPC, and 13.68 for QTL5 and defoliation AUDPC.

**Fig. 5:**
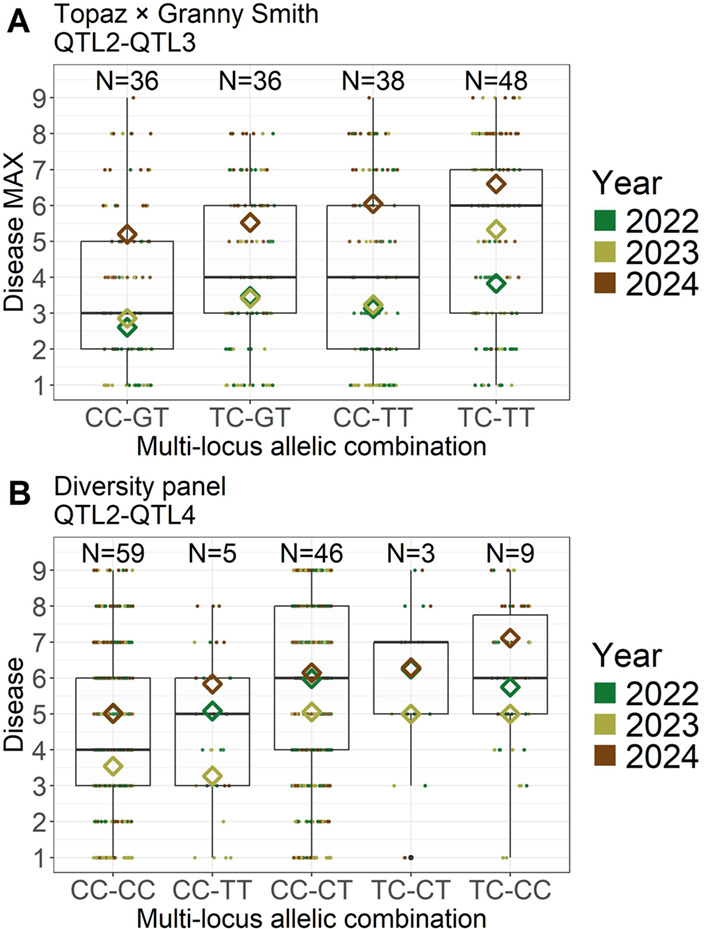
Boxplots showing the distribution of traits across multi-locus allelic combinations and years. Boxes represent the interquartile range, with the median indicated by a horizontal line. Whiskers extend to 1.5 times the interquartile range. The jittered points show the phenotypic values for each genotype, colored according to the year of measurement. Diamonds represent the mean value for each year across genotypes. The number of genotypes for each multi-locus allelic combination is shown above the corresponding box. The multi-locus allelic combinations are presented in order of increasing mean values estimated across years. **A** Boxplots for disease MAX of the ‘Topaz’ × ‘Granny Smith’ cross and the multi-locus allelic combinations derived from QTL2 and QTL3. **B** Boxplots for the disease trait of the diversity panel and the multi-locus allelic combinations derived from QTL2 and QTL4

Both QTL2 and QTL3 were associated with disease MAX in the ‘Topaz’ × ‘Granny Smith’ cross, and differences between multi-locus allelic combinations derived from QTL2 and QTL3 were observed for disease MAX (Fig. 5A). Across years, the mean phenotypic values for the disease MAX were 3.55 for the multi-locus allelic combination CC-GT, 4.14 for TC-GT and CC-TT, and 5.26 for TC-TT, the latter corresponding to a widespread infection (grade 5). A likelihood ratio test for the mixed-effects model, which included a fixed effect of the multi-locus allelic combination × year interaction and a random effect of genotype, revealed a significant effect of the tested interaction (*p* = 0.00755).

QTL2 and QTL4 were both associated with the disease MAX trait in different crosses, and the SNPs associated with these QTLs were present in the genomic dataset of the diversity panel, enabling the construction of multi-locus allelic combinations within the diversity panel (Fig. 5B). The mean phenotypic values across years for the disease trait were 4.53 for CC-CC, 4.77 for CC-TT, 5.74 for CC-CT, 5.86 for TC-CT, and 5.98 for TC-CC. A likelihood ratio test of the multi-locus allelic combination × year interaction was not significant (*p* = 0.08672), indicating weak evidence for the interaction effect (0.05 < *p* ≤ 0.10). When tested separately, the effects of multi-locus allelic combination and year were both significant (*p* = 0.01058 and *p* < 0.00001, respectively).

### Location of the quantitative trait loci in the diversity panel

SNPs associated with QTL1, QTL2, and QTL4 were available in the genomic dataset for the diversity panel. For QTL5, the associated SNP was not available in the genomic dataset for the diversity panel, and a haplotype block was assigned to QTL5 based on the physical position of the associated SNP within the block. For QTL3, neither an SNP nor a directly overlapping haplotype block could be identified. In this case, the SNP with the next lowest *p*-value that could be assigned to a haplotype block on the same chromosome was selected instead (GDsnp01761), located at 40.45 Mb. The physical position was 0.36 Mb downstream of the original associated SNP for QTL3. The number of identified haplo-alleles was 19 for QTL3 and 17 for QTL5. The haplotype blocks for QTL3 and QTL5 were composed of six SNPs each (QTL3: AX-115459416, AX-115459417, AX-115459418, AX-115459419, AX-115459420, AX-105179063; QTL5: AX-115311535, AX-115311536, AX-115559875, AX-115559873, AX-115559872, AX-115185714).

### Variance components

**Fig. 6:**
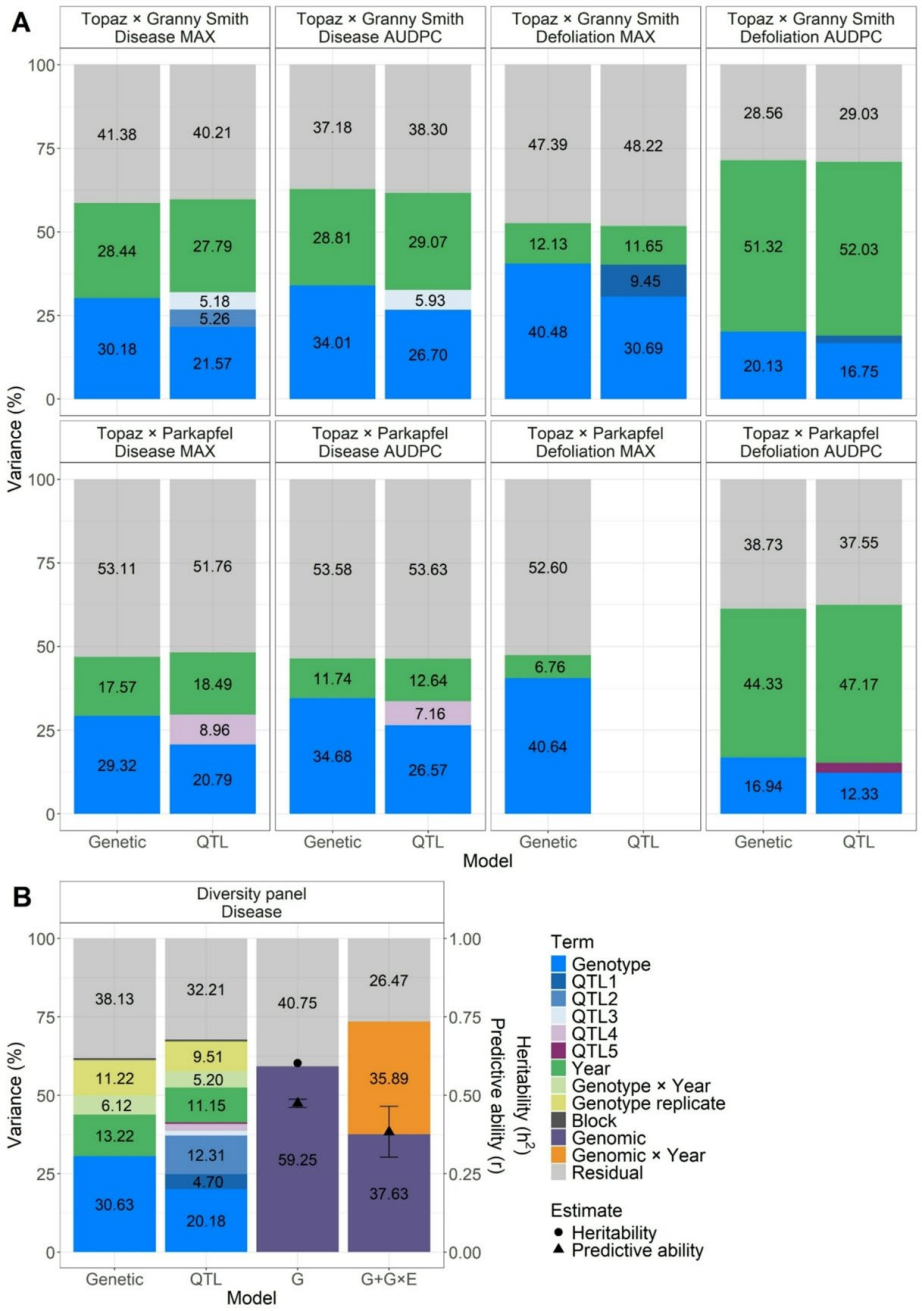
Phenotypic variance decomposition, genomic heritability and predictive ability. **A** Phenotypic variance decomposition for summary traits and two crosses, using the genetic model and the QTL model (if at least one QTL was identified). The QTL model estimated QTL effects based on individual SNPs. **B** Phenotypic variance decomposition for the disease trait from the diversity panel, using genetic, QTL, G, and G + G×E models. The QTL model estimated QTL effects using individual SNPs or haplotype blocks when SNPs were unavailable. Estimates of genomic heritability and average predictive ability for the disease trait are represented by different shapes. For predictive ability, error bars indicate the standard deviation around the mean. **AB** QTLs with explained variance ($\:{\sigma\:}^{2}$) below 3% are not numerically labeled (panel A: QTL1 $\:{\sigma\:}^{2}=2.18\%$, QTL5 $\:{\sigma\:}^{2}=2.96\%$, panel B: QTL3 $\:{\sigma\:}^{2}=1.51\%$, QTL4 $\:{\sigma\:}^{2}=2.21\%$, and QTL5 $\:{\sigma\:}^{2}=0.46\%$). The block effects in panel B explained 0.68% and 0.58% variance for the genetic and QTL model, respectively. The legend shown in panel B also applies to panel A

In the genetic model, phenotypic variance decomposition revealed that genotype and year effects contributed more substantially to the total explained variance in the ‘Topaz’ × ‘Granny Smith’ cross compared to the ‘Topaz’ × ‘Parkapfel’ cross for most summary traits (Fig. 6A). Among the summary traits, defoliation MAX showed the highest contribution of the genotype effect and the lowest contribution of the year effect in the genetic model across crosses. In contrast, defoliation AUDPC exhibited the lowest contribution of the genotype effect and the highest contribution of the year effect. The QTL model indicated that each of the five QTLs accounted for less than 10% of the total variance.

Using the genetic model to study the diversity panel, the genetic effect for the disease trait accounted for 30.63% of the total variance, comparable to the combined contributions of year, genotype × year interaction, genotype replicate, and block effects of 31.24% (Fig. 6B). In the QTL model, the highest contribution among the QTLs of 12.31% was observed for QTL2. QTL1 showed a contribution of 4.70%, while QTL3 accounted for 1.51%, QTL4 for 2.21%, and QTL5 for 0.46% of the total variance.

When all SNP markers of the diversity panel were used in the G model to explain variance in the disease trait, the resulting genomic effects accounted for 59.25% of the total variance (Fig. 6B). Based on the proportion of phenotypic variance explained by these genomic effects, the estimated genomic heritability of 0.61 indicated that the disease trait is subject to a moderately high level of genetic control. Using the G + G×E model, genomic main effects accounted for 37.63% of the total phenotypic variance, while genomic × year interaction effects explained an additional 35.89%. Average predictive ability was estimated at 0.47 (standard deviation of 0.01) for the G model, and 0.38 (standard deviation of 0.08) for the G + G×E model.

## Discussion

### Seasonal effects drive trait variation

Crosses between the susceptible cultivar ‘Topaz’ and the partially resistant genotypes ‘Granny Smith’ and ‘Parkapfel’ enabled the estimation of phenotypic values spanning the full spectrum of resistance, from complete absence of symptoms to high susceptibility (Fig. 1). The diversity panel exhibited similar trait distributions to both crosses, suggesting uniform disease pressure across all studied populations and years. Only in 2023 was a slightly lower proportion of highly diseased individuals observed within the diversity panel compared to 2022 and 2024, coinciding with reduced precipitation in June and September (Online Resource 4). Generally, AB severity increased over successive years, indicating progressive disease spread as the orchards became older. This temporal dynamic likely contributed to the low interannual correlations of disease and defoliation traits assessed at different timepoints per year between 2024 and the two preceding years (Fig. 2). Nonetheless, within each year and cross, disease and defoliation traits measured at different timepoints were highly correlated (Fig. 2), supporting the use of maximum scores to represent within-year disease severity. Across years, substantial year effects were revealed using variance decomposition of the summary traits based on the genetic model (Fig. 6), confirming the need to remove the year effects through the estimation of BLUEs. The BLUEs provided phenotypic values adjusted for seasonal effects for subsequent QTL mapping and genomic prediction, which was in contrast to previous genomic research that could not account for seasonal effects because only single-year data were available (Noh et al. 2020). Our multi-year analysis also revealed a pronounced genotype × year interaction (Fig. 6), indicating that the same genotypes exhibited different susceptibility levels across years. This pattern is expected when disease spread is heterogeneous, underscoring the importance of repeated disease assessments over multiple years.

### Genetic link between disease and defoliation

Premature leaf drop is linked to reduced carbohydrate production, which leads to weakened growth and lower fruit yield (Sagong et al. 2011). As one of the major symptoms of AB disease, defoliation was assessed in our study using an adapted scoring scale originally applied for disease evaluation (Lateur and Populer 1994; Patocchi et al. 2009). Although disease and defoliation traits were highly correlated (Fig. 2), no QTLs were shared between disease and defoliation traits in our analysis (Fig. 3). However, QTL1 identified in the ‘Topaz’ × ‘Granny Smith’ cross, which explained 9.45% of the phenotypic variance for defoliation, showed an effect of 4.70% on disease in the diversity panel (Fig. 6), supporting a genetic link between defoliation and disease scores. Individuals exhibiting high disease scores but low defoliation may experience fewer physiological consequences of carbohydrate depletion compared to those that shed diseased leaves more rapidly. Such genotypes could be valuable as breeding material in the future.

### Polygenic trait architecture

Several studies have investigated the genetic architecture of resistance to AB (Richter et al. 2025; Zhou et al. 2012; Noh et al. 2020). Using a transcriptomic approach, Zhou et al. (2012) identified multiple defense-related genes in the resistant Chinese commercial cultivar ‘Qinguan’. A GWAS conducted for AB resistance, which used resistance scores collected under natural field conditions in a Korean germplasm collection, identified significant QTLs on chromosomes 3, 9, 10 and 17 (Noh et al. 2020). More recently, Richter et al. (2025) conducted a GWAS using germplasm from a German gene bank and cultivar collection, which was artificially inoculated with AB in the laboratory using a detached-leaf assay, and detected significant associations on chromosomes 3, 12, 13, 15 and 16. In the present study, QTL mapping revealed partially overlapping results, with loci detected on chromosomes 3, 5, 6, 10, and 11.

On chromosome 10, Noh et al. (2020) identified a locus at 40.31 Mb, which is in close proximity to our QTL3 at 40.09 Mb in the ‘Topaz’ × ‘Granny Smith’ cross. While Noh et al. (2020) assessed disease severity during a single growing season, our disease MAX trait similarly reflects disease severity, though across multiple years. Given the alignment in phenotyping approach (natural field conditions and direct severity measures) and the close physical proximity of the loci, the findings suggest that both studies have identified the same genetic region associated with AB resistance on chromosome 10. Nevertheless, the effect of QTL3 remained difficult to quantify in the diversity panel, as the SNP associated with QTL3 was not available in the diversity panel dataset due to incompatibility between SNP arrays for this marker (Howard et al. 2021b), and no haplotype block could be directly assigned to this SNP. The nearest SNP was located 0.36 Mb downstream, and its corresponding haplotype block explained 1.51% of the phenotypic variance in the diversity panel, compared with ~ 5% explained by QTL3 in the ‘Topaz’ × ‘Granny Smith’ cross (Fig. 6). Despite the high resolution of the genomic dataset (303,237 SNPs) in the diversity panel, additional genotyping would be required to reliably assess the effect of QTL3.

On chromosome 3, previous studies reported associated loci at 15.40 Mb on the same reference genome used in our study (Noh et al. 2020) and 14.42 Mb on an alternative reference genome (Richter et al. 2025), with the latter explaining 41.04% of the phenotypic variance. In contrast, our identified QTL1 on chromosome 3 was located further upstream at 5.18 Mb and explained 9.45% of the phenotypic variance for the defoliation MAX trait in the ‘Topaz’ × ‘Granny Smith’ cross, and 4.70% of the disease trait variance in the diversity panel (Fig. 6). The difference between the physical locations of QTL1 and the previously reported locus cannot be attributed to insufficient SNP coverage in the latter region. This is supported by the presence of 13 SNPs between 14.00 and 16.00 Mb in the genomic data for the ‘Topaz’ × ‘Granny Smith’ cross, nine of which were heterozygous in the ‘Granny Smith’ parent segregating at QTL1. Due to the substantial physical distance between previously reported loci and our QTL1, it is unlikely that our locus on chromosome 3 corresponds to those identified in earlier studies.

The remaining loci identified on chromosomes 5, 6, and 11 have not been previously presented in the literature and thus represent novel information about the genetic architecture of resistance to AB. All the loci reported in this study, individually explaining a relatively small proportion of phenotypic variance (up to ~ 12% each, Fig. 6), support earlier findings that AB resistance is a polygenic trait (Richter et al. 2025; Zhou et al. 2012; Noh et al. 2020). Indeed, the genomic effects estimated using 303 K SNP markers in the G model, when seasonal (i.e., year) effects were removed, explained an increased proportion of variance compared to that captured by the genotype and QTL effects in the QTL model (explained variance of 59.25% for the G model and 41.37% for the QTL model, Fig. 6). These results suggest that rather than relying on a limited number of QTLs with minor effects, genome-wide approaches may offer a more effective strategy for identifying and selecting germplasm with enhanced AB resistance.

### Localization of identified quantitative trait loci for apple blotch relative to quantitative trait loci for other diseases in apple

If QTLs associated with different apple diseases are located close together in the genome, this may indicate either pleiotropy, where a single gene influences multiple diseases, or tight linkage, where distinct loci are inherited together. However, systematic comparisons of QTLs across studies are challenging due to the limited reliability of arbitrary physical distance thresholds. In the absence of a standardized cutoff, applying a ≤ 0.50 Mb distance threshold provides a pragmatic compromise, helping to avoid criteria that are either too stringent or too permissive when identifying potentially overlapping QTLs. Within the ≤ 0.50 Mb threshold, we identified a previously reported QTL on chromosome 3 associated with apple scab resistance (Bénéjam et al. 2021), located 0.34 Mb from QTL1. On chromosome 5, two QTLs for fire blight resistance have been reported near QTL2, at distances of 0.08 Mb (Le Roux et al. 2010) and 0.35 Mb (Bénéjam et al. 2021). A combination of additional genetic and functional evidence is needed to confirm whether these QTLs are closely located or refer to a single gene with pleiotropic effect.

### Allelic contributions of quantitative trait loci across mapping and diversity panels

In a QTL mapping experiment targeting disease resistance, it is generally expected that the resistant parent is heterozygous at the QTL, and that the susceptible parent is homozygous for the susceptibility allele. As a result, the subset of the progeny heterozygous at the QTL is expected to exhibit greater resistance than the subset of progeny that is homozygous for the susceptibility allele. In the ‘Topaz’ × ‘Granny Smith’ cross, however, the homozygous allelic combinations at the associated markers, identical to the combinations carried by ‘Topaz’ at QTL1 and QTL2, showed increased resistance compared to the heterozygous allelic combination (Fig. 4; Online Resource 3). Interestingly, the same allelic combinations carried by ‘Topaz’ were also associated with reduced disease symptoms in the diversity panel (Online Resource 5). Although the favorable allelic combinations at QTL1 and QTL2 each had detectable effects, they were insufficient to confer a high level of AB resistance in ‘Topaz’ and the studied germplasm. Thus, the resistance observed in ‘Granny Smith’ likely relies on additional QTLs beyond these two. Indeed, in the mapping population of the ‘Topaz’ × ‘Granny Smith’ cross, the parent ‘Granny Smith’ provided the resistance allele found in QTL3. While the heterozygous allelic combination containing this resistance allele exhibited a clear effect within the mapping population (Fig. 6A), it showed only a very weak effect in the diversity panel (Fig. 6B). This may be due to the absence of the associated SNP in the genomic dataset of the diversity panel, or the limited presence of the underlying allele within the wider germplasm of the diversity panel.

For QTL4 and QTL5, identified in the ‘Topaz’ × ‘Parkapfel’ cross, increased resistance was also observed in the homozygous allelic combination at the associated marker, identical to the combination found in the resistant parent ‘Parkapfel’, while the parent heterozygous at the QTL was the susceptible parent ‘Topaz’ (Fig. 4; Online Resource 3). Additionally, these QTL were not detected in the other cross, despite ‘Topaz’ being a common parent between the two crosses, suggesting that the genetic background provided by ‘Parkapfel’ was necessary to reveal the heterozygosity of ‘Topaz’ at these QTLs. Among the two QTLs, QTL4 displayed a stronger effect in the mapping population, while the effect of QTL5 was low (Fig. 6A). However, in the diversity panel, the results indicated a non-additive genetic effect at QTL4, with the heterozygous allelic combination being more susceptible than either of the homozygous classes (Online Resource 5).

The fact that increased resistance was associated with homozygous allelic combinations at four out of five QTLs indicates that complete dominance is unlikely to be the mode of gene action at these loci. This atypical pattern for resistance loci may indicate the involvement of susceptibility genes, where loss or reduction of function confers enhanced resistance, and emphasizes the need for further functional validation to clarify the underlying genetic mechanisms.

### Approaches to selection

The development of mapping populations and subsequent QTL mapping in this study were conducted with the aim of identifying loci that could enable marker-assisted selection for AB resistance. Due to the absence of resistant cultivars (Wöhner and Emeriewen 2019; Hinrichs-Berger and Müller 2013), the mapping populations were derived from crosses involving partially resistant parental genotypes. The limited and partly contradictory information on cultivar resistance to AB has complicated the selection process. For example, the reports on the resistance level of the parental genotype ‘Granny Smith’ vary widely, ranging from disease-free (Sharma et al. 2011), to resistant with < 1% infected leaves (Noh et al. 2020), to moderately susceptible with 65% infected leaves (Rather et al. 2017). Additional cultivars with reduced susceptibility to AB may be found within the diversity panel, as several genotypes showed disease score BLUEs across years not exceeding ~ 3, with the cultivar ‘Grauer Hordapfel’ (MUNQ 1343) reaching the lowest BLUE of 1.67 (Online Resource 1). Moreover, potential sources of partial resistance for breeding may be identified among highly diseased individuals that exhibit low defoliation, because high disease scores do not necessarily correspond to severe defoliation (Fig. 1). However, a high disease load in non-defoliated trees may increase inoculum levels in the orchard, potentially leading to more severe outbreaks in subsequent seasons. In addition, the timing of defoliation is critical, where retention of leaves until after harvest may help maintain sufficient tree vitality for production. Nevertheless, the effects on productivity and vitality were not assessed in this study due to the young age of trees in the biparental populations.

The future identification of additional cultivars exhibiting strong resistance could enable the development of new mapping populations, potentially leading to the discovery of novel QTLs with high effects suitable for breeding applications using marker-assisted selection. Nevertheless, a significant effect of the multi-locus allelic combinations derived from QTL2 and QTL4 was found in the diversity panel (Fig. 5B), supporting the initiation of marker-assisted selection for reduced AB susceptibility using markers associated with QTL2 and QTL4.

In the absence of high-effect QTLs, genomic selection offers a viable alternative to marker-assisted selection. This is primarily because genomic prediction models use genome-wide marker information, enabling them to capture a greater proportion of phenotypic variance attributable to numerous small-effect QTLs (Heffner et al. 2009). Consistent with this, the genomic prediction models evaluated in our study explained a substantially larger proportion of phenotypic variance compared to mixed-effects models incorporating only individual QTL effects (Fig. 6).

The predictive ability of both genomic prediction models tested in this study was moderate relative to a broad range of apple traits previously evaluated using similar prediction approaches (Jung et al. 2022). The predictive ability, and consequently the reliability of genomic predictions, could likely be enhanced by expanding the training population, represented here by only 122 genotypes of the diversity panel. As shown by Hickey et al. (2014), achieving high prediction accuracy often requires phenotyping thousands of individuals, particularly when the training and target populations are only distantly related. Nevertheless, even the moderate predictive ability observed in this study has the potential to accelerate genetic gain (Heffner et al. 2010). The availability of the diversity panel dataset, along with the trained genomic prediction models, establishes a foundation for the first application of modern genomics-assisted selection technologies in breeding for resistance to AB.

## Conclusion

This study provides new insights into the phenotypic and genetic basis of AB resistance under natural field conditions. By integrating data from biparental populations and a diversity panel, we demonstrated that AB resistance is influenced by strong seasonal effects and characterized by a polygenic architecture. While the identified QTLs were of small effect and therefore not immediately applicable for marker-assisted selection, they expand the current understanding of resistance loci, including the discovery of several novel regions. The moderate yet promising predictive ability of the genomic prediction models suggests that they could improve breeding efficiency in the absence of high-effect QTLs. The use of BLUEs to adjust for environmental variability and the establishment of a diversity panel with trained genomic prediction models provide a foundation for implementing genomics-assisted selection strategies in apple breeding. Future efforts should focus on enlarging the training population, identifying resistant germplasm, and refining phenotyping strategies to further improve predictive ability and accelerate the development of AB-resistant cultivars.

## Supplementary Information

Below is the link to the electronic supplementary material.

Supplementary Material 1

Supplementary Material 2

Supplementary Material 3

Supplementary Material 4

Supplementary Material 5

## Data Availability

The genomic datasets analyzed in this study, along with the phenotypic datasets generated for the segregating populations, are available on Zenodo at https://doi.org/10.5281/zenodo.19589792.
