# Supplementary material for: QTL mapping and genomic prediction of resistance to apple blotch (Diplocarpon coronariae)
Source: Mol Breed. 2026 May 2;46(5):44. doi: 10.1007/s11032-026-01666-5 (PMC13135599; doi:10.1007/s11032-026-01666-5)
Supplement: Supplementary file 4 — Supplementary Material 4 [file 11032_2026_1666_MOESM4_ESM.pdf]

Molecular Breeding

QTL mapping and genomic prediction of resistance to apple blotch (*Diplocarpon coronariae*)

Michaela Jung[1], Bettina Hänni[1,2], Hélène Muranty[3], Andrea Patocchi[1]
[1]Agroscope, Mueller-Thurgau-Strasse 29, 8820 Waedenswil, Switzerland
[2]Fructus, Mueller-Thurgau-Strasse 29, 8820 Waedenswil, Switzerland
[3]Univ Angers, Institut Agro, INRAE, IRHS, SFR QuaSaV, F-49000 Angers, France

Corresponding authors: Michaela Jung, michaela.jung@agroscope.admin.ch; Andrea Patocchi, andrea.patocchi@agroscope.admin.ch

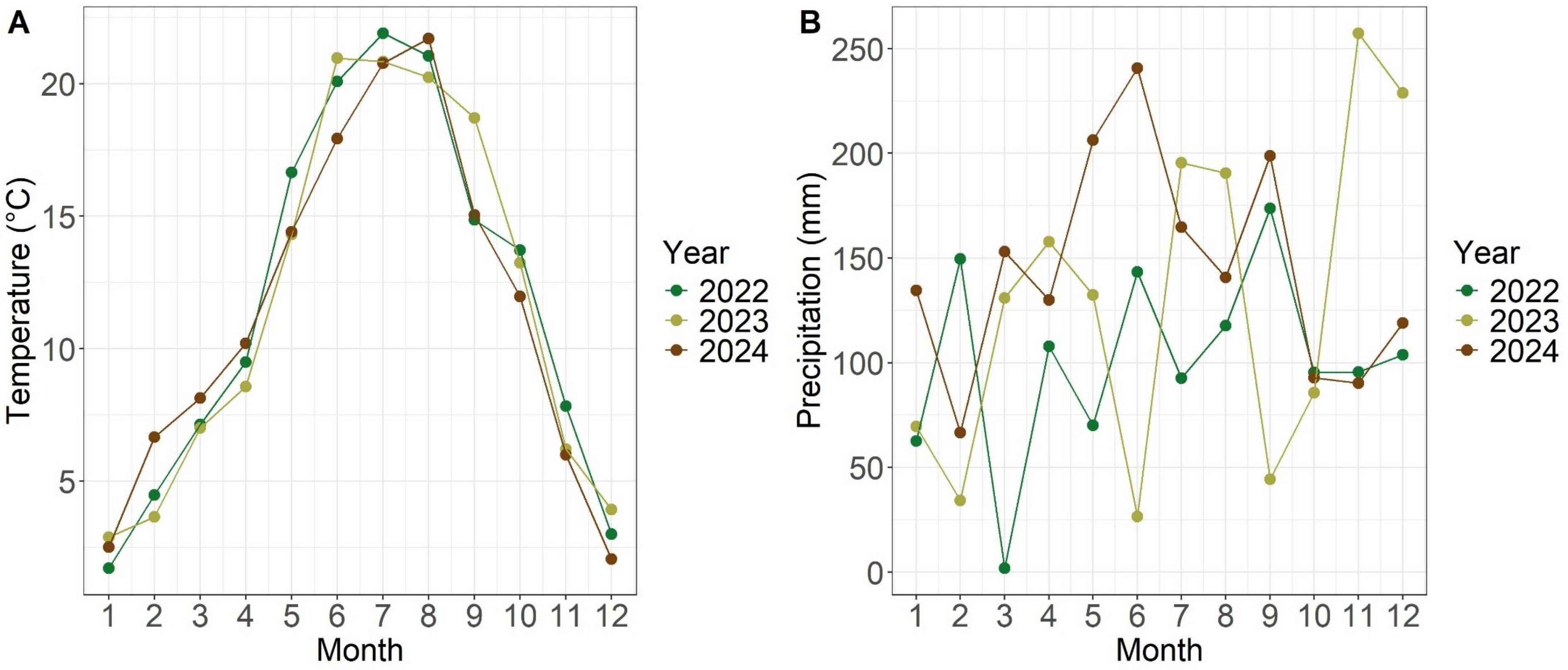

Online Resource 4: Variables from the weather station near the studied orchards. **A** Average monthly temperature at 2 m above soil level. **B** Sum of monthly precipitation.
