# Supplementary material for: QTL mapping and genomic prediction of resistance to apple blotch (Diplocarpon coronariae)
Source: Mol Breed. 2026 May 2;46(5):44. doi: 10.1007/s11032-026-01666-5 (PMC13135599; doi:10.1007/s11032-026-01666-5)
Supplement: Supplementary file 5 — Supplementary Material 5 [file 11032_2026_1666_MOESM5_ESM.pdf]

Molecular Breeding

QTL mapping and genomic prediction of resistance to apple blotch (*Diplocarpon coronariae*)

Michaela Jung[1], Bettina Hänni[1,2], Hélène Muranty[3], Andrea Patocchi[1]
[1]Agroscope, Mueller-Thurgau-Strasse 29, 8820 Waedenswil, Switzerland
[2]Fructus, Mueller-Thurgau-Strasse 29, 8820 Waedenswil, Switzerland
[3]Univ Angers, Institut Agro, INRAE, IRHS, SFR QuaSaV, F-49000 Angers, France

Corresponding authors: Michaela Jung, michaela.jung@agroscope.admin.ch; Andrea Patocchi, andrea.patocchi@agroscope.admin.ch

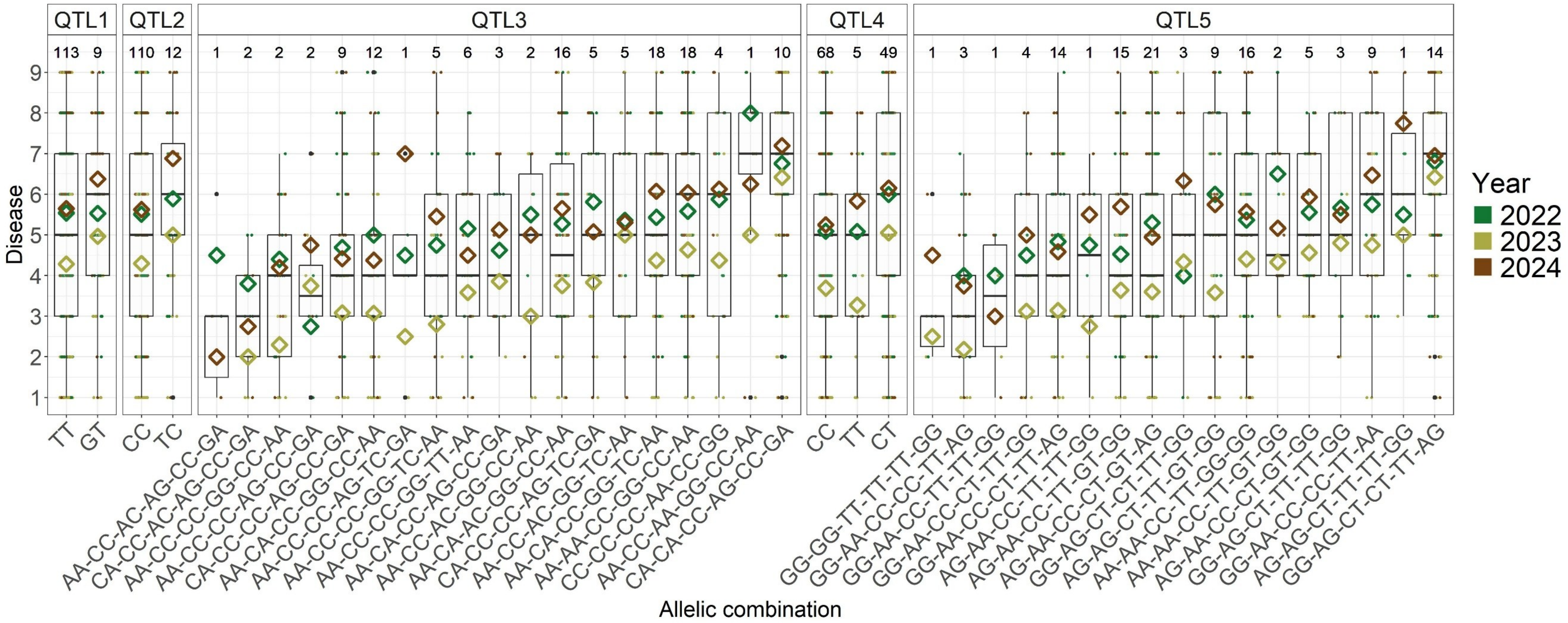

Online Resource 5: Boxplots showing the distribution of the disease trait in the diversty panel across years and allelic combinations for five QTLs. For each QTL, the allelic combinations are presented in order of increasing mean values across years. The number of genotypes for each allelic combination is shown above the corresponding box. Boxes represent the interquartile range, with the median indicated by a horizontal line. Whiskers extend to 1.5 times the interquartile range, and black points outside this range represent outliers. The jittered points show the phenotypic values for each genotype, colored according to the year of measurement. Diamonds represent the mean value for each year across genotypes.
